# Validity and reliability of the Noor Evidence-Based Medicine Questionnaire: A cross-sectional study

**DOI:** 10.1371/journal.pone.0249660

**Published:** 2021-04-22

**Authors:** Mohd Noor Norhayati, Zanaridah Mat Nawi

**Affiliations:** Department of Family Medicine, School of Medical Sciences, Health Campus, Universiti Sains Malaysia, Kelantan, Malaysia; Iwate Medical University, JAPAN

## Abstract

**Background:**

Evidence-based medicine (EBM) is a widely accepted scientific advancement in clinical settings that helps achieve better, safer, and more cost-effective healthcare. However, presently, validated instruments to evaluate healthcare professionals’ attitude and practices toward implementing EBM are not widely available. Therefore, the present study aimed to determine the validity and reliability of a newly developed knowledge, attitude, and practice (KAP) questionnaire on EBM for use among healthcare professionals.

**Methods:**

The Noor Evidence-Based Medicine Questionnaire was tested among physicians in a government hospital between July and August 2018. Exploratory factor analysis and internal consistency reliability-based Cronbach’s alpha statistic were conducted.

**Results:**

The questionnaire was distributed among 94 physicians, and 90 responded (response rate of 95.7%). The initial number of items in the KAP domains of the Noor Evidence-Based Medicine Questionnaire were 15, 17, and 13, respectively; however, two items in the practice domain with communalities <0.25 and factor loadings <0.4 were removed. The factor structure accounted for 52.33%, 66.29%, and 55.39% of data variance in the KAP domains, respectively. Cronbach’s alpha values were 0.81, 0.81, and 0.84 for KAP domains, respectively, indicating high reliability.

**Conclusions:**

This questionnaire can be used to evaluate the knowledge, attitudes, and behaviour of healthcare professionals toward EBM. Future testing of this questionnaire among other medical personnel groups will help expand the scope of this tool.

## Background

Evidence-based medicine (EBM) is the deliberate, transparent, and judicious use of existing best evidence in decision making about individual patient treatment [1,2]. It relies on three pillars, namely, (i) individual clinical expertise (healthcare professionals), (ii) patient values and desires, and (iii) best research available [3]. Hence, EBM is an essential component of medical practice and constitutes explicitly stated practice [4]. Both knowledge and attitude of healthcare professionals play a vital role in determining EBM implementation [5]. However, a large gap in attitude, information, and practices related to EBM has been reported globally, resulting in incomplete EBM application in real-world scenarios [6,7].

In Malaysia, the implementation of evidence-based practice is low because of limited awareness, knowledge, attitude, and resources. Many healthcare practitioners use non-evidence-based sources and access the same through new approaches such as texting. These findings were derived from a qualitative study among medical officers and family medicine specialists that identified five themes related to EBM among healthcare professionals: (i) doctors predominantly regarded EBM as statistics, research, and guidelines; (ii) reactions to EBM were overwhelmingly negative; (iii) doctors relied on consultants, colleagues, and their guidance, and non-evidence-based internet sources for information; (iv) information sources were accessed using novel methods such as mobile applications; and (v) there were several barriers to evidence-based practice, including physician-, EBM-, patient- and system-related factors, such as inadequacies in knowledge, attitude, management support, time, and access to authentic information sources [8].

Several questionnaires are available for evaluating EBM awareness among healthcare professionals, such as Fresno, Berlin, Baum, McColl, and EBM Questionnaire [9–17]. The most commonly used questionnaire developed by McColl et al. [18] evaluates self-reported awareness, attitudes, and barriers toward EBM [16,19]. Baum’s Questionnaire also measures attitude toward EBM and self-reported EBM capabilities among healthcare professionals [15], whereas the Berlin questionnaire measures knowledge and skills related to EBM [13].

The Fresno test is considered the best questionnaire. It evaluates both competency and skills related to EBM and, thereby, serves as a reliable and accurate method for detecting instruction impact [9]. However, the assessment of inter-rater reliability, internal consistency, and discrimination were intimately dependent on the population which has taken the test and are all likely to be lower with a more homogenous group of evidence-based medicine learners [20]. While the adapted Fresno test is relevant to rehabilitation professionals and has removed some statistical questions [21]. The Assessing Competency in EBM (ACE) tool, in addition to the Fresno test, is also a reliable and validated instrument for evaluating EBM competency among healthcare practitioners. The ACE method offers a novel method of assessment as it evaluates user performance in four main steps; however, its application across various patient scenarios remains limited [10]. Notably, the use of multiple EBM tests has resulted in the incongruous assessment of available EBM domains in a sample because of the heterogeneity of items on these tools [12].

Despite these research tools’ availability, new questionnaires should be developed, adapted and tested. The purpose of the development of the current questionnaire is to assess the depth of knowledge, direction of attitude, and practice of EBM among healthcare professionals. This questionnaire’s scoring system will be based on each item to produce valid overall and cut-off scores irrespective of the population in which it is being tested. As it is necessary to have a tool to identify gaps and improve EBM implementation in clinical practice, the development, and validation of psychometrically robust instruments is crucial. Therefore, the present study aimed to determine the validity and reliability of a newly developed knowledge, attitude, and practice questionnaire on EBM for healthcare professionals.

## Methods

### EBM questionnaire

The development of the Noor EBM questionnaire began with a literature search on EBM to ensure content quality. A literature search was undertaken in Medline to ensure good content of the scale; keywords such as “evidence-based medicine”, “evidence-based practice”, “healthcare” “doctors”, “questionnaire” and “scale” were used.

Using a modified Delphi method, the questionnaires were structured to encourage research team members to participate in the final round to clarify the problems and present arguments that explain their views [22]. Four experts, including a public health physician, a family medicine specialist, an evidence-based medicine expert, and a biostatistician, were involved in the development process. Each item is dealt with in detail to ensure that it is appreciated in the same way by all respondents. It avoided unclear content in one item or a double-barreled item, complicated or ambiguous words, different thoughts or notions.

Two sets of questionnaires were prepared for each item to ask for a similar context but differently. With ten respondents, including experts in the field and healthcare professionals, the 45-item questionnaires underwent cognitive debriefing. For consistency, appropriateness, and significance, each item was evaluated. The wordings of several items have been updated accordingly.

The Noor EBM questionnaire originally comprised knowledge, attitude, and practice domains with 15, 17, and 13 items. All three domains required responses on a five-point Likert scale with the knowledge and attitude domains using the Strongly Agree = 5/Agree = 4/Neutral = 3/Disagree = 2/Strongly Disagree = 1 scale, and the practice domain using the Always = 5/Often = 4/Sometimes = 3/Seldom = 2/Never = 1 scale. Reverse scoring was used for negatively worded items.

Total scores were calculated for each domain, and each total raw score was transformed into “percent score” by dividing the raw score with the maximum score and multiplying it by 100. It was done to appreciate differences better, as the scale would then range from 0 to 100. The results of this questionnaire can be expressed in outcome scores and categories, and the percent score for each domain can be categorized based on Bloom’s cut-off point (60%–79%) [23]; this method has been used in other studies as well [24,25]. Scores within this range were defined as moderate knowledge, neutral attitude, and fair level of practice. In contrast, scores above this cut-off point were considered as a high level of knowledge, positive attitude, and a good level of practice. Scores below this cut-off point were equated with low knowledge, negative attitude, and poor practice level.

### Participants

In this cross-sectional study, construct validity and reliability were evaluated among randomly selected physicians at the Hospital Sungai Buloh between July to August 2018. Physicians registered with the national medical council and working in a government hospital in Selangor, Malaysia, were recruited. House officers were excluded. Sample size calculation was based on the attitude domain’s responses (17 items), and a subject item ratio of 1 to 5 [26] was applied. A sample size of 94 subjects was obtained, assuming a 10% non-response rate.

Convenient sampling was applied wherein the researcher explained the present study and distributed an informed consent form. Once the participant understood and consented to join the study, they were given the self-administered questionnaire and were encouraged to self-complete the EBM scale. The questionnaire was written in English, as it is a global language and used to train doctors in most medical schools. Upon completion, questionnaires were checked for completeness of responses, and the participants were thanked for their co-operation. Participation in the present study was not expected to lead to any potential or foreseeable risk.

### Ethics approval and consent to participate

The research proposal was approved by the Research and Ethics Committee of the Universiti Sains Malaysia (USM/JEPeM/18040195) and the National Research Medical Registration (NMRR-18-349-40727). All patients gave written informed consent before answering the questionnaire. Confidentially of the data were maintained through anonymity and presented as grouped data.

### Statistical analyses

Exploratory factor analysis (EFA) and internal consistency reliability-based Cronbach’s alpha statistic were used to estimate the questionnaire’s construct validity and reliability. Certain items were carefully eliminated without compromising content validity based on the set parameters. The principal axis factoring extraction method with Promax rotation was applied to produce a solution with the best simple structure. Items were treated as continuous responses to allow evaluation of dimensionality (number of factors) of the items. To meet this assumption, the Kaiser–Meyer–Olkin (KMO) statistic was calculated, and a cut-off value of >0.7 was used to ensure data suitability for factor analysis. Furthermore, Bartlett’s test of sphericity with a *p* value of <0.05, indicating good correlation among items, was mandated. To determine the number of extracted factors, Eigenvalues of >1.0, parallel analysis, and scree plot inspection was conducted [27]. Communalities >0.25 and factor loadings >0.4 were considered acceptable [28]. For internal consistency reliability, a Cronbach’s alpha coefficient >0.7 was considered acceptable. All statistical analyses were conducted using the IBM SPSS Statistics version 24. A *p* value of <0.05 was considered statistically significant.

## Results

In the present study, questionnaires were distributed to 94 clinicians, and a response rate of 95.7% was recorded. Participants included 32 men (35.6%), 23 specialized clinicians (25.6%), 65 Malays (72.2%), and 34 married respondents (37.8%). The average age (standard deviation, SD) of the cohort was 31.8 years (3.97; range 26–46 years), and median (interquartile range) experience in healthcare was two years (3.0; range 1–10).

### Knowledge domain on EBM

The mean score for individual items in the knowledge domain ranged from 2.9 to 4.4 (SD range 0.59–1.07; Table 1). Item-level descriptive statistics of the 15 items in the knowledge domain showed that item K1, “Evidence-based medicine involves the process of critically appraising research findings as to the basis for clinical decisions” had the highest mean (SD) score at 4.4 (0.59).

**Table 1:** Psychometric properties of the knowledge domain on EBM.

Factor	Item	Description	% of variance	Mean (SD^a^)	Factor loading	CITC^b^
Factor 1	K1	a. Evidence-based medicine involves the process of critically appraising research findings as the basis for clinical decisions.	30.24	4.4 (0.59)	0.54	0.36
	K8	a. Four essential components structured in the PICO format (Patient or problem, Intervention, Comparison, Outcome) will make a good clinical question.		3.9 (0.63)	0.61	0.43
	K9	a. Evidence-based medicine improves clinicians’ understanding of research methodology.		4.1 (0.59)	0.60	0.55
	K11	a. Evidence-based medicine can be practiced in situations where there is doubt about any aspect of clinical management.		4.0 (0.70)	0.57	0.50
	K12	a. Improving access to summaries of evidence is appropriate to encourage evidence-based practice.		4.0 (0.64)	0.50	0.45
	K13	b. The increasing number of systematic reviews that are applicable to general practice can be found in the Cochrane Library.		3.8 (0.74)	0.77	0.57
	K14	a. Difficulty in understanding statistical terms is the major setback in applying evidence-based medicine.		4.1 (0.74)	0.59	0.49
	K15	a. Application of evidence-based practice is cost-effective to healthcare system.		3.6 (0.91)	0.54	0.08
Factor 2	K2	b. Evidence-based medicine focuses on the best current available research without considering clinical experience.	13.88	3.5 (0.96)	0.69	0.58
	K3	a. Evidence-based medicine is suitable for making decisions about care of patients rather than for policymaking.		2.9 (1.07)	0.79	0.43
	K4	a. Patients’ preferences should be prioritized over clinicians’ preferences in making clinical decisions.		3.0 (0.98)	0.53	0.27
	K5	b. Evidence-based medicine improves clinical management by using evidence from meta-analysis only.		3.2 (0.94)	0.64	0.43
	K6	a. Evidence-based medicine does not help to promote self -directed learning.		3.9 (0.76)	0.42	0.56
	K10	b. Clinicians who practice evidence-based medicine become less critical in using data in systemic reviews.		3.5 (0.88)	0.52	0.45
Factor 3	K7	a. Meta-analysis is superior to case–control studies in evidence-based medicine.	8.22	3.8 (0.81)	0.52	0.35

^a^ Standard deviation.

^b^ Corrected item-total correlation.

EFA for the knowledge domain initially yielded a KMO of 0.76, and Bartlett’s Test of Sphericity was significant (P < 0.001). None of the items was removed due to low communalities or low factor loading, and all 15 items were retained with factor loading ranging from 0.42 to 0.78. Factor analysis of the 15 items yielded a three-factor structure that explained 52.33% of the data variance. However, factor 3 comprised only one item. The overall Cronbach’s alpha score for the knowledge domain was 0.81, and the mean (SD) total score for the 15 items was 55.6 (6.31).

### Attitude domain on EBM

The mean scores for individual items in the attitude domain ranged from 2.8 to 4.4 (SD range 0.50–1.18; Table 2). Item-level descriptive statistics of the 17 items in the attitude domain showed that item A15, “I think it is mandatory for physicians to continuously update their knowledge to deliver efficient patient care” had the highest mean (SD) score at 4.4 (0.66).

**Table 2:** Psychometric properties of the attitude domain on EBM.

Factor	Item	Description	% of variance	Mean (SD^a^)	Factor loading	CITC^b^
Factor 1	A1	I believe that evidence-based medicine is a threat to good clinical practice.	31.37	4.1 (0.62)	0.53	0.38
	A2	I believe practicing evidence-based medicine improves patient health outcome.		4.2 (0.54)	0.72	0.50
	A3	I am keen to learn evidence-based medicine if given the opportunity.		4.2 (0.57)	0.81	0.46
	A4	I am ready to practice evidence-based medicine in my work.		4.0 (0.66)	0.69	0.60
Factor 2	A5	I feel that research findings are very important in my day-to-day management of patients.	12.03	4.1 (0.58)	0.60	0.51
	A11	I feel that access to databases is vital in obtaining journals on evidence-based medicine.		4.2 (0.64)	0.77	0.40
	A15	I think it is mandatory for physicians to continuously update their knowledge to deliver efficient patient care.		4.4 (0.66)	0.65	0.50
	A16	I am interested in receiving education materials on evidence-based medicine as they relate to various topics.		4.2 (0.59)	0.53	0.53
	A17	I think that educational interventions and incorporating formal teaching of evidence-based medicine at medical education are very important.		4.1 (0.50)	0.35	0.53
Factor 3	A8	I am convinced that applying evidence-based medicine in clinical practice increases the effectiveness of my work.	8.30	4.0 (0.68)	0.92	0.42
	A9	I feel confident in managing patients with evidence-based medicine.		4.09 (0.57)	0.62	0.48
	A13	I feel that practicing evidence-based medicine would produce better health practitioners.		4.10 (0.56)	0.44	0.44
Factor 4	A6	I feel that evidence-based medicine is of limited value in general practice because management in primary care requires less scientific evidence.	7.70	3.77 (0.85)	0.45	0.53
	A7	I believe that years of clinical experience is more valuable than evidence-based medicine.		3.26 (0.80)	0.62	0.30
	A14	I often feel burdened whenever needing to use evidence-based medicine in practice.		3.38 (0.89)	0.53	0.46
Factor 5	A10	I am certain that understanding the basic mechanism of disease is sufficient for good clinical practice.	6.89	2.81 (1.18)	0.66	0.02
	A12	I feel that reading the conclusions of a systemic review is adequate for clinical practice.		3.13 (0.96)	0.60	0.36

^a^ Standard deviation.

^b^ Corrected item-total correlation.

EFA for the attitude domain had an initial KMO value of 0.74, and Bartlett’s Test of Sphericity was significant (P < 0.001). None of the items was removed due to low communalities or low factor loading, and all 17 items were retained with factor loading ranging from 0.44 to 0.92. The factor analysis of the 17 items yielded a five-factor structure that explained 66.29% of the variance in data. However, factors 3, 4, and 5 comprised only three or two items. The overall Cronbach’s alpha statistic for the attitude domain was 0.81, and the mean (SD) total score for the 17 items was 66.2 (6.06).

### Practice domain on EBM

The mean score for individual items in the practice domain ranged from 1.6 to 3.7 (SD range 0.80–1.26; Table 3). Item-level descriptive statistics of the 13 items in the practice domain showed that item P6, “I consider patient’s wishes in the decision making process,” had the highest mean (SD) score at 3.7 (0.92).

**Table 3:** Psychometric properties of practice domain for EBM.

Factor	Item	Description	% of variance	Mean (SD^a^)	Factor loading	CITC^b^
Factor 1	P1	I apply evidence-based medicine in practice.	40.74	3.3 (0.80)	0.58	0.56
	P2	I use multiple search engines for systemic review.		3.2 (0.95)	0.65	0.63
	P3	I search for evidence-based medicine material from published journals only.		3.2 (1.00)	0.58	0.53
	P6	I consider the patient’s wishes in the decision making process.		3.7 (0.92)		0.09
	P7	I use evidence-based medicine for answering the questions in a clinical setting.		3.5 (0.92)	0.72	0.54
	P8	I only use the computer provided at my workplace rather than a smartphone to search for online databases.		3.0 (1.03)		0.37
	P9	I join continuous medical education for an update regarding evidence-based medicine.		3.4 (0.92)	0.54	0.47
	P10	I promote evidence-based practice to my colleagues at the workplace.		3.0 (1.18)	0.68	0.72
	P11	I share my knowledge of evidence-based medicine with my colleagues.		3.3 (1.08)	0.76	0.65
	P12	I am involved in the development of clinical practice guidelines.		1.6 (1.10)	0.61	0.49
	P13	I usually translate a clinical question into a form that can be answered from the literature.		2.1 (1.26)	0.70	0.59
Factor 2	P4	I do not have enough time to study evidence-based medicine.	14.66	2.8 (1.12)	0.77	0.26
	P5	I cannot practice evidence-based medicine due to limitations of the management that I can offer to patients in clinic settings.		3.13 (1.10)	0.72	0.26

^a^ Standard deviation.

^b^ Corrected item-total correlation.

During EFA, the initial KMO statistic for the practice domain was 0.77, and Bartlett’s Test of Sphericity was significant (P < 0.001). Two items, P6 (I consider the patient’s wishes in the decision making process) and P8 (I only use the computer provided at my workplace rather than a smartphone to search for online databases) with communalities <0.25 and factor loadings <0.4 were removed, and their removal was judged as not affecting content validity. Thus, the final number of items in the practice domain was 11, with factor loading ranging from 0.43 to 0.99. Factor analysis of the 11 items yielded a two-factor structure that explained 55.39% of the data variance, and factor 2 comprised only two items (P4, P5). The overall Cronbach’s alpha statistic for the practice domain was 0.84. The mean (SD) total score for the 11 items was 32.5 (7.09).

## Discussion

The advent of EBM and the growing role of the evidence from these studies in the decision making process has necessitated the assessment of relevant competencies among healthcare providers. This Noor EBM questionnaire was intended for use among healthcare professionals, including medical officers and specialist physicians, and hence adequately represents the target population during the evaluation of psychometric parameters in the tool.

The results presented here show that the Noor EBM questionnaire performed well. The reliability of the individual domains was significantly greater than the suggested value for Cronbach’s alpha [29], and this high reliability implies a high degree of confidence in our findings. This is related to the small random error point, which did not exceed 10% to 20%, suggesting that random fluctuations in test results that are defined in classical psychometric test theory do not reduce the value of alpha in this case [30].

The EFA for the practice domain led to the removal of two items, P6 and P8. In P6, “I consider patient’s wishes in the decision making process,” the word “wishes” is rather vague and out of context, which may have resulted in participants rejecting it as a legitimate item. For item P8 (I only use the computer provided at my workplace rather than a smartphone to search for online databases), its rejection may be due to improper selection of search devices in the healthcare delivery setting.

Measurements obtained for the Noor EBM questionnaire’s individual items suggest a relatively high level of similarity among study participants in all three domains, i.e., knowledge, attitude, and practice. Furthermore, we found a minimal variation (low SD range), suggesting high uniformity in measured traits. Similar findings were seen with mean scores for individual items on the questionnaire, implying the absence of a “floor’ and “ceiling” effect, i.e., a narrow range between extreme mean values for all three domains (knowledge: 2.9 to 4.4; attitude: 2.81 to 4.36; and practice: 1.57 to 3.71).

In the present study, the average score of items in the knowledge and attitude domains ranged between 3 and 4, suggesting that the physicians were either “neutral to” or “agreed to” the statements. Although such a score may reflect “unsure” to “reasonably adequate” knowledge and neutral to positive attitudes, it also indicates that greater focus must be laid on increasing EBM knowledge and positive reinforcement of attitudes. The average score of items in the practice domain ranged from about 2 to 4, suggesting that EBM practice ranged from “seldom” to “practice.” This finding indicates that, in the future, special consideration should be paid to behaviour and competency in EBM awareness programs among healthcare professionals.

Although studies have shown positive attitudes toward EBM among healthcare professionals [5,19,31–36], there is evidence of insufficient experience [19,31,33]. One study reported that only a small percentage (<10%) of medical trainee specialists could correctly state the hierarchy of best types of evidence [7]. Many healthcare practitioners have also reported that they did not use it although they had heard about EBM [37].

The most significant impediments to EBM adoption have been listed as differences in human disease (61.0%), followed by a lack of hospital/department funding (39.8%) [5]. A review of 28 papers on barriers to EBM implementation found that the level of understanding, expertise, and evidence-based performance was less than 50.0%. This was due to lack of equipment, resources, and research methodology skills. Additionally, the degree of familiarity with EBM-related terminology was low (44.2%), and textbooks were regarded as the most important source of knowledge [38].

The Noor EBM questionnaire evaluated healthcare professionals’ knowledge, attitudes, and behaviour toward EBM. It has been tested in a survey among primary-care practitioners in Selangor, Malaysia; the results are published in a separate manuscript. The findings can be used to detect and correct obstacles in the workplace that hinder EBM practice. Thus, the questionnaire can also be used for developing training programs, as it can highlight areas that require particular emphasis or focus. The Noor EBM questionnaire’s modular nature is undoubtedly an advantage as individual domains can be used in isolation, based on the needs of the study. Future studies may translate the Noor EBM questionnaire’s English version into the Malay language or any other local languages.

As with any instrument, the Noor EBM questionnaire has some weaknesses. One of them is the inability to determine the use of other pieces of non-scientific information. Clinical decisions based on non-scientific facts, personal experience, practice, or intuitions contradict the principle of EBM. Future testing of the Noor EBM questionnaire in other groups of medical specialists will expand the applicability of this tool. Retesting the questionnaire’s robustness in other populations to produce an adapted shorter version and the degree of agreement with this original is suggested for future research.

Another limitation of the present study is the lack of convergent validity analysis; this psychometric property could not be evaluated because of the lack of a standard tool for evaluating EBM. Second, we developed the items without imposing certain subdomains to test for construct validity. However, the factors that emerged were too inadequate as individual subdomains due to the limited number of items. The separation of items into subdomains did not reveal a specific representational theme. Third, confirmatory factor analysis was not conducted on a different set of samples to confirm the unidimensional construct of the findings. However, scholars have argued that EFA is more appropriate when analyzing new scales [39].

## Conclusions

Psychometric analysis of the Noor EBM questionnaire confirms that this tool is of high quality. The questionnaire may be used to evaluate competency, attitude, and behaviour toward EBM among healthcare professionals.

## Supporting information

S1 File Noor Evidence-Based Medicine Questionnaire. (PDF)

S2 File Enago certificate. (SAV)

## Abbreviations

EBM: evidence-based medicine
EFA: exploratory factor analysis
KMO: Kaiser–Meyer–Olkin
