# Supplementary material for: Validity and reliability of the Noor Evidence-Based Medicine Questionnaire: A cross-sectional study
Source: PLoS One. 2021 Apr 22;16(4):e0249660. doi: 10.1371/journal.pone.0249660 (PMC8061909; doi:10.1371/journal.pone.0249660)
Supplement: S1 File — (PDF) [file pone.0249660.s001.pdf]

Noor Evidence-Based Medicine Questionnaire

The following items are regarding knowledge, attitude and practice towards evidence-based medicine. Indicate with a tick (√) the best alternative for each item, and take care not to skip any items.

	**Knowledge of evidence-based medicine**	Strongly Agree	Agree	Neutral	Disagree	Strongly Disagree
1	Evidence-based medicine involves the process of critically appraising research findings as to the basis for clinical decisions.					
2	Evidence-based medicine focuses on the best current available research without considering clinical experience.					
3	Evidence-based medicine is suitable for making decisions about the care of patients rather than for policymaking.					
4	Patients’ preferences should be prioritized over clinicians’ preferences in making clinical decisions.					
5	Evidence-based medicine improves clinical management by using evidence from meta-analysis only.					
6	Evidence-based medicine does not help to promote self - directed learning.					
7	Meta-analysis is superior to case-control studies in evidence-based medicine.					
8	Four essential components structured in the PICO format (Patient or problem, Intervention, Comparison, Outcome) will make a good clinical question.					
9	Evidence-based medicine improves clinicians’ understanding of research methodology.					
10	Clinicians who practice evidence-based medicine become less critical in using data in systemic reviews.					
11	Evidence-based medicine can be practiced in situations where there is doubt about any aspect of clinical management.					
12	Improving access to summaries of evidence is appropriate to encourage evidence-based practice.					
13	The increasing number of systematic reviews that are applicable to general practice can be found in the Cochrane Library.					
14	Difficulty in understanding statistical terms is the major setback in applying evidence-based medicine.					
15	Application of evidence-based practice is cost-effective to the healthcare system.					

	**Attitude on evidence-based medicine**	Strongly Agree	Agree	Neutral	Disagree	Strongly Disagree
1	I believe that evidence-based medicine is a threat to good clinical practice.					
2	I believe practicing evidence-based medicine can improve patient health outcome.					
3	I am keen to learn evidence-based medicine if given the opportunity.					
4	I am ready to practice evidence-based medicine in my work.					
5	I feel that research findings are very important in my day-to-day management of patients.					
6	I feel that evidence-based medicine is of limited value in general practice because management in primary care requires less scientific evidence.					
7	I believe that years of clinical experience is more valuable than evidence-based medicine.					
8	I am convinced that applying evidence-based medicine in clinical practice increases the effectiveness of my work.					
9	I feel confident managing patients with evidence-based medicine.					
10	I am certain that understanding the basic mechanisms of disease is sufficient for good clinical practice.					
11	I feel that access to databases is vital in obtaining journals on evidence-based medicine.					
12	I feel that reading the conclusions of a systematic review is adequate for clinical practice.					
13	I feel that practicing evidence-based medicine would produce better health practitioners.					
14	I often feel burdened whenever needing to use evidence-based medicine in practice.					
15	I think it is mandatory for physicians to continuously update their knowledge to deliver efficient patient care.					
16	I am interested in receiving educational materials on evidence-based medicine as they relate to various topics.					
17	I think that educational interventions and incorporating formal teaching of evidence-based medicine at medical education are very important.					

	**Practice of evidence-based medicine**	Always	Often	Sometimes	Seldom	Never
1	I apply evidence-based medicine in practice.					
2	I use multiple search engines for systematic review.					
3	I search for evidence-based medicine material from published journals only.					
4	I do not have enough time to study evidence-based medicine.					
5	I cannot practice evidence-based medicine due to limitations of the management that I can offer to patients in clinic settings.					
6	I use evidence based-medicine for answering the questions in a clinical setting.					
7	I join continuous medical education for an update regarding evidence-based medicine.					
8	I promote evidence-based practice to my colleagues at the workplace.					
9	I share my knowledge of evidence-based medicine with my colleagues.					
10	I am involved in the development of clinical practice guideline.					
11	I usually translate a clinical question into a form that can be answered from the literature.					

Noor Evidence-Based Medicine Questionnaire

Scoring of items

	**Knowledge of evidence-based medicine**	Strongly Agree	Agree	Neutral	Disagree	Strongly Disagree
1	Evidence-based medicine involves the process of critically appraising research findings as to the basis for clinical decisions.	5	4	3	2	1
2	Evidence-based medicine focuses on the best current available research without considering clinical experience.	1	2	3	4	5
3	Evidence-based medicine is suitable for making decisions about the care of patients rather than for policymaking.	1	2	3	4	5
4	Patients' preferences should be prioritized over clinicians' preferences in making clinical decisions.	1	2	3	4	5
5	Evidence-based medicine improves clinical management by using evidence from meta-analysis only.	1	2	3	4	5
6	Evidence-based medicine does not help to promote self - directed learning.	1	2	3	4	5
7	Meta-analysis is superior to case-control studies in evidence-based medicine.	5	4	3	2	1
8	Four essential components structured in the PICO format (Patient or problem, Intervention, Comparison, Outcome) will make a good clinical question.	5	4	3	2	1
9	Evidence-based medicine improves clinicians' understanding of research methodology.	5	4	3	2	1
10	Clinicians who practice evidence-based medicine become less critical in using data in systemic reviews.	1	2	3	4	5
11	Evidence-based medicine can be practiced in situations where there is doubt about any aspect of clinical management.	5	4	3	2	1
12	Improving access to summaries of evidence is appropriate to encourage evidence-based practice.	5	4	3	2	1
13	The increasing number of systematic reviews that are applicable to general practice can be found in the Cochrane Library.	5	4	3	2	1
14	Difficulty in understanding statistical terms is the major setback in applying evidence-based medicine.	5	4	3	2	1
15	Application of evidence-based practice is cost-effective to the healthcare system.	5	4	3	2	1

	**Attitude on evidence-based medicine**	Strongly Agree	Agree	Neutral	Disagree	Strongly Disagree
1	I believe that evidence-based medicine is a threat to good clinical practice.	1	2	3	4	5
2	I believe practicing evidence-based medicine can improve patient health outcome.	5	4	3	2	1
3	I am keen to learn evidence-based medicine if given the opportunity.	5	4	3	2	1
4	I am ready to practice evidence-based medicine in my work.	5	4	3	2	1
5	I feel that research findings are very important in my day-to-day management of patients.	5	4	3	2	1
6	I feel that evidence-based medicine is of limited value in general practice because management in primary care requires less scientific evidence.	1	2	3	4	5
7	I believe that years of clinical experience is more valuable than evidence-based medicine.	1	2	3	4	5
8	I am convinced that applying evidence-based medicine in clinical practice increases the effectiveness of my work.	5	4	3	2	1
9	I feel confident managing patients with evidence-based medicine.	5	4	3	2	1
10	I am certain that understanding the basic mechanisms of disease is sufficient for good clinical practice.	1	2	3	4	5
11	I feel that access to databases is vital in obtaining journals on evidence-based medicine.	5	4	3	2	1
12	I feel that reading the conclusions of a systematic review is adequate for clinical practice.	1	2	3	4	5
13	I feel that practicing evidence-based medicine would produce better health practitioners.	5	4	3	2	1
14	I often feel burdened whenever needing to use evidence-based medicine in practice.	1	2	3	4	5
15	I think it is mandatory for physicians to continuously update their knowledge to deliver efficient patient care.	5	4	3	2	1
16	I am interested in receiving educational materials on evidence-based medicine as they relate to various topics.	5	4	3	2	1
17	I think that educational interventions and incorporating formal teaching of evidence-based medicine at medical education are very important.	5	4	3	2	1

	Practice of evidence-based medicine	Always	Often	Sometimes	Seldom	Never
1	I apply evidence-based medicine in practice.	5	4	3	2	1
2	I use multiple search engines for systematic review.	5	4	3	2	1
3	I search for evidence-based medicine material from published journals only.	5	4	3	2	1
4	I do not have enough time to study evidence-based medicine.	1	2	3	4	5
5	I cannot practice evidence-based medicine due to limitations of the management that I can offer to patients in clinic settings.	1	2	3	4	5
6	I use evidence based-medicine for answering the questions in a clinical setting.	5	4	3	2	1
7	I join continuous medical education for an update regarding evidence-based medicine.	5	4	3	2	1
8	I promote evidence-based practice to my colleagues at the workplace.	5	4	3	2	1
9	I share my knowledge of evidence-based medicine with my colleagues.	5	4	3	2	1
10	I am involved in the development of clinical practice guideline.	5	4	3	2	1
11	I usually translate a clinical question into a form that can be answered from the literature.	5	4	3	2	1
